# Selection of Reference Genes for Normalization of Gene Expression After Exposure of Human Endothelial and Epithelial Cells to Hypoxia

**DOI:** 10.3390/ijms26041763

**Published:** 2025-02-19

**Authors:** Juliane Hannemann, Lena Schmidt-Hutten, Jannik Hannemann, Fiona Kleinsang, Rainer Böger

**Affiliations:** Institute of Clinical Pharmacology and Toxicology, University Medical Center Hamburg-Eppendorf, 20246 Hamburg, Germany; lena.schmidt-hutten@web.de (L.S.-H.); boeger@uke.de (R.B.)

**Keywords:** quantitative real-time polymerase chain reaction, hypoxia, endothelial cells, gene expression, housekeeping gene

## Abstract

The selection of a stably expressed reference gene is a critical step for the quantitation of gene expression by qRT-PCR. We tested the stability of expression of nine putative reference genes in normoxia and hypoxia in four different human cell types: coronary (HCAECs) and pulmonary endothelial cells (HPAECs), EA.hy926 endothelial cells, and A549 alveolar epithelial cells. Cells were cultured in normoxic and hypoxic conditions for up to 72 h. Total RNA was isolated and used for qRT-PCR. Stability of expression was assessed by calculating the coefficient of variation of the cycle threshold (Ct CV) by pairwise comparison of ΔCt values, and by the NormFinder algorithm. A final rank was calculated for each gene. Finally, we analyzed *VEGFA* expression by using *GAPDH* or the optimal candidate reference gene found in this study. Gene expression was variable between cell lines and experimental conditions. The most stable reference gene across all cell lines was *TBP*, followed by *RPLP1* and *RPL13A*. *VEGFA* expression was significantly upregulated by 4-fold in hypoxia when using *TBP* as reference, whilst this result was insignificant when *GAPDH* was used. The selection of a stably expressed reference gene is a critical step for the generation of reliable and reproducible data in gene expression studies. The most appropriate reference gene may vary in different cell lines and experimental conditions; it should be chosen individually for each experimental set-up.

## 1. Introduction

The quantification of messenger RNA (mRNA) expression is an important method to gain better insight into cellular regulatory networks, helping us to understand underlying pathophysiological mechanisms. Quantitative real-time polymerase chain reaction (qRT-PCR) [1] is the most widely used technique for the quantification of gene transcription. Nevertheless, standardization of this method is critical, and results differ depending on the methods of RNA isolation and processing, the protocol used for reverse transcription, and the amount of starting material. Since mRNA represents only the minor part of total RNA mass, suitable controls are crucial for correct read-out and interpretation of the data obtained by any qRT-PCR experiment [2].

The state-of-the-art method to standardize qRT-PCR experiments is the use of reference or housekeeping genes for normalization, allowing the quantification of mRNA of the transcripts of interest relative to that of the housekeeping gene. To enable this, the chosen internal control genes need to be stably expressed in the cells and/or in the experimental conditions of the actual experiment [3]. Often, however, housekeeping genes are chosen based on their known ubiquitous expression and the general assumption that this expression is constant even in different conditions. However, it has been known for years that expression of many of the classical, often-used housekeeping genes may vary in different tissues, cell types, or experimental conditions [4,5], and it is widely accepted that optimal reference genes need to be determined a priori for a specific experimental setup [6].

In particular, hypoxia represents a challenge for gene expression analyses, since a number of well-described housekeeping genes show strong differential regulation between normoxic and hypoxic conditions. In addition, this may vary in different cell types. Differential expression of two often-used housekeeping genes, glyceraldehyde-3-phosphate dehydrogenase (*GAPDH*) and β-actin (*ACTB*), in hypoxia has repeatedly been reported in vitro and in vivo: *GAPDH* belongs to the least stably expressed genes in human retinal endothelial cells [7] and bladder cancer cells [8] in hypoxia; its protein levels also increase to 3–4-fold in rat alveolar epithelial cells after 18 h of hypoxia [9]. Published data suggest that *GAPDH* is directly upregulated by an interaction between the hypoxia inducible factors-1α and -2α through a 5′ hypoxic regulatory element [10]. Similar results have been reported for *ACTB*, which is another high abundance gene, but might also be differentially expressed in hypoxia, depending on the type of cells under investigation [11].

Whilst many recent publications have described the identification of suitable reference genes to investigate cancer-related hypoxia [8,11,12,13,14,15], less data are available on suitable reference genes to compare gene expression in primary endothelial cells in normoxia and hypoxia. This is important, however, since the endothelium is the major cell type involved in the regulation of hypoxic vasodilation in the systemic circulation and hypoxic vasoconstriction in the pulmonary circulation, respectively [16]. It is therefore of the utmost importance to select the optimal reference gene for the specific experimental setup prior to the experiments.

The aim of the present study was to identify optimal endogenous control genes to investigate gene expression in primary human coronary and pulmonary endothelial cells (HCAECs, HPAECs) in comparison to an immortalized human endothelial cell line (EA.hy926), as well as an immortalized human lung alveolar epithelial cell line (A549), in conditions of normoxia and hypoxia.

## 2. Results

### 2.1. Expression Profiles of Putative Reference Genes in Normoxia and Hypoxia

**Figure 1:**
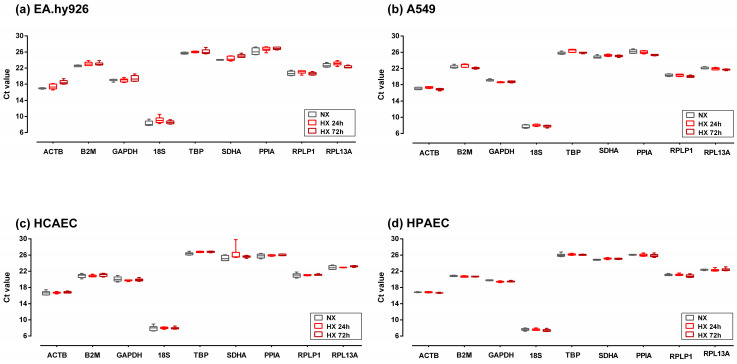
Cycle threshold (Ct) values of the nine candidate reference genes used in this study in EA.hy926 cells (**a**), A549 cells (**b**), HCAEC (**c**), and HPAEC (**d**). Cells were cultured in normoxia (NX) or in hypoxia for 24 h (HX 24 h) or 72 h (HX 72 h). Box plots represent the interquartile range (IQR, 25th–75th percentiles). Horizontal lines represent the median, and whiskers indicate the minimum and maximum values. Abbreviations: A549, immortalized human alveolar epithelial cells; EA.hy926, immortalized human umbilical vein endothelial cells; HCAECs, primary human coronary artery endothelial cell; HPAECs, primary human pulmonary artery endothelial cells; HX, hypoxia; NX, normoxia; abbreviations of gene names are detailed in Table S1.

Ct values for each putative reference gene were similar across all four cell lines. With a Ct value of 8.0 ± 0.6, *18S* was the most abundantly expressed gene in all cell lines. The lowest expression levels throughout all cell lines were observed for TBP (26.2 ± 0.4), *PPIA* (26.1 ± 0.5), and *SDHA* (25.1 ± 0.8) (Figure 1).

When comparing gene expression in normoxia versus hypoxia, there were differential responses of the various genes between cell lines. For example, *ACTB* expression remained stable in hypoxic HCAECs and HPAECs, whilst it increased in EA.hy926 cells and slightly decreased after 72 h of hypoxia in A549 cells (Figure 1). By contrast, *TBP*, *RPL13A*, and *RPLP1* showed the least variation in gene expression in response to hypoxia.

### 2.2. Expression Stability of Putative Reference Genes in Normoxia and Hypoxia in Each Cell Line

**Figure 2:**
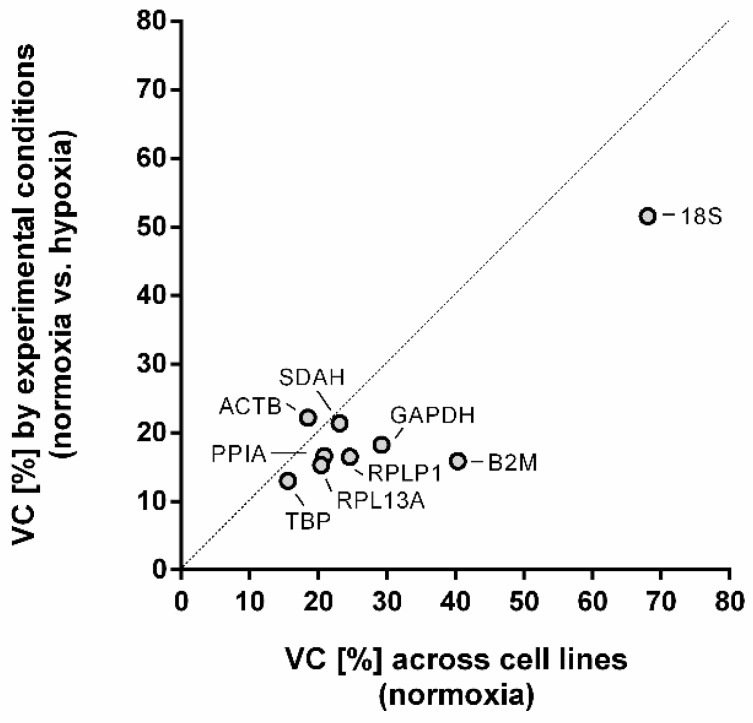
Plot of variation coefficients (VC; filled dots) for each putative reference gene, showing variability across the four cell lines studied (HCAECs, HPAECs, EA.hy926, and A549) on the *x*-axis, and variability between normoxia and hypoxia (24 h and 72 h) on the *y*-axis. The dashed line marks the line of equality between variability according to both components.

Variation of gene expression of the putative reference genes was affected by two components: (a) differences between the four cell lines, and (b) differences between normoxia and hypoxia. In Figure 2, the coefficient of variation for each gene according to both components is plotted. The graph shows that the highest variability was observed for *18S* and *B2M*, whilst *TBP* showed the lowest variability for both dimensions. In general, variability between cell lines was slightly higher than variability caused by oxygen availability, except for *ACTB* which showed higher variability due to experimental conditions. As variability due to hypoxia showed little variation between 24 and 72 h (Figure 2), further analyses were performed by comparing NX with HX (24 h and 72 h combined). Accordingly, Table 1 lists coefficients of variation of Ct values (Ct CV) in normoxia and hypoxia (24 h and 72 h) for all putative reference genes in each of the four cell lines analyzed.

Next, we analyzed the expression stability of the candidate reference genes by pairwise ΔCt comparison of genes within each cell line according to Silver et al. [17]. The mean SD reflects the expression stability of the respective reference gene, with a low mean SD value indicating a more stable expression. Genes were ranked from low to high mean SD (Table 2). Some genes appeared to be stably expressed in one cell line, but showed more variability in another. For example, *ACTB* had a low mean SD in A549 cells (0.276, rank 2), but a high mean SD in EA.hy926 cells (0.764, rank 9). *RPLP1* showed low variation in A549, HCAECs, and HPAECs (mean SD: 0.290, 0.310, and 0.226, respectively), but high variation in EA.hy926 (mean SD 0.555). *PPIA* (0.590, rank 1) and *TBP* (0.604, rank 2) were the most stably expressed putative reference genes across all cell lines (Table 2).

**Figure 3:**
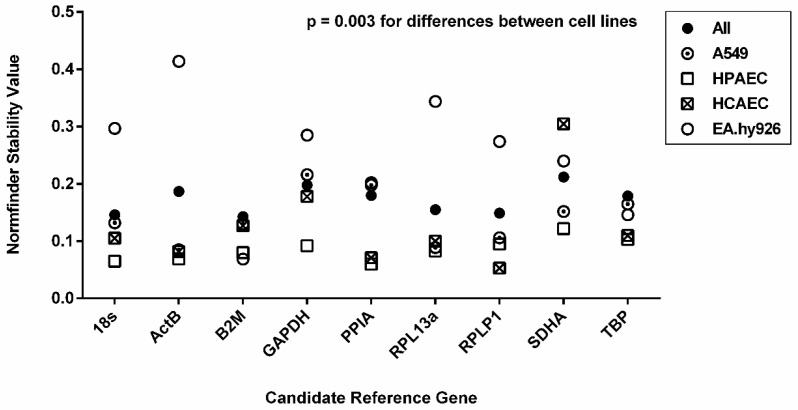
Stability values calculated by the NormFinder algorithm. Stability values were calculated for each cell line separately, differentiating between three biological groups (NX, HX 24 h, HX 72 h), respectively. Using relative quantities, the algorithm calculates intra- and intergroup variation, resulting in lower stability values for candidate genes with higher stability in gene expression.

Finally, the NormFinder algorithm was used to analyze gene expression stability. An overview of the ranking based on the NormFinder results in the different cell lines is given in Figure 3.

In HPAECs, the three top-ranking genes (*PPIA*, *18S* and *ACTB*) showed very similar stability values of 0.060, 0.065 and 0.069, respectively. In HCAECs, the top-ranking gene was *RPLP1* (stability value 0.053) followed by *PPIA* and *ACTB* on ranks two and three (stability values 0.071 and 0.082, respectively). In EA.hy926 cells, the top ranking gene (*B2M*) had a stability value of 0.069, followed by other genes with considerably higher NormFinder values indicating less stable expression (*TBP*, 0.103; *PPIA*, 0.202). In A549 cells, the two top-ranking genes (*ACTB*, 0.085 and *RPL13A*, 0.089) showed similar gene expression stabilities, followed by *RPLP1* with a slightly higher expression variation (0.106). Comparing the stability values of candidate genes between the four cell lines, HCAECs, HPAECs and A549 showed similar expression stability for several genes, whereas EA.hy926 had clearly higher stability values for all candidate reference genes, except for *B2M* and *TBP* (Figure 3).

### 2.3. Finding the Optimal Reference Gene Across All Four Cell Lines

The variation analyses of putative reference genes were also performed collectively for all four cell lines. The results of NormFinder analysis for all four cell lines combined are shown in Figure 3. The four candidate reference genes with lowest stability values were *B2M*, *18S*, *RPLP1*, and *RPL13A* (stability values, 0.143, 0.146, 0.149, and 0.155, respectively), whereby stability values of these reference genes appeared to be similar.

As each of the expression stability analyses produced slightly different results, we used a combination of all methods to find the most suitable reference gene for our studies. We calculated the sum rank score of each candidate reference gene in each cell line, and we performed the same calculation for all cell lines combined; the results are shown in Table 3. The top-ranking reference gene for studying the effects of hypoxia across all cell lines analyzed was *TBP*, followed by *RPLP1* and *PPIA*, whilst all other genes turned out to be less stably expressed (Table 3).

### 2.4. Identification of the Most Stably Expressed Reference Gene in Combinations of Two Cell Lines

We performed the analyses for three different combinations of two cell lines each to address a variety of possible research topics: (a) We compared EA.hy926 cells and HCAECs, i.e., a commonly used, immortalized human umbilical vein endothelial cell line and primary human coronary arterial endothelial cells, both being derived from the systemic circulation. *RPLP1* was the most stable reference gene for this comparison. (b) We next compared HCAECs and HPAECs, i.e., primary human endothelial cells from the systemic and pulmonary circulation in NX and HX, respectively. *B2M* and *RPLP1* turned out to be the most stable reference genes for this comparison. (c) Finally, we compared epithelial and endothelial cells from the lung, i.e., the A549 human alveolar epithelial cell line and HPAECs. For this comparison, *TBP* was the most stably expressed reference gene. The ranks of putative reference genes for each of these comparisons are listed in Table 4; detailed results from the Ct CV, pairwise ΔCt, and NormFinder analyses are presented in Table S2.

### 2.5. Effects of Reference Gene Selection on the Analysis of VEGFA Gene Expression in Hypoxia

**Figure 4:**
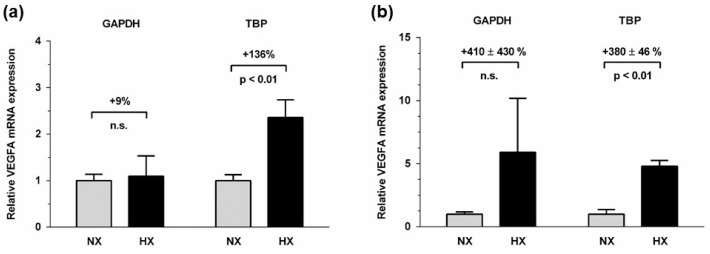
Relative mRNA expression of *VEGFA* normalized to the respective reference gene indicated. Relative mRNA expression was calculated using the ΔΔCt method, relative mRNA expression in normoxia was set to 1. (**a**) *VEGFA* mRNA expression after 24 h of incubation in normoxia (NX) or hypoxia (HX) of A549 cells, by using either *GAPDH* (left panel) or *TBP* (right panel) as reference gene; (**b**) *VEGFA* mRNA expression after 72 h of incubation in normoxia (NX) or hypoxia (HX) of EA.hy926 cells, by using either *GAPDH* (left panel) or *TBP* (right panel) as the reference gene. Abbreviations: n.s., not significant.

To exemplify the impact of reference gene selection on gene expression analyses in hypoxia, we compared *VEGFA* expression when normalized to a commonly used reference gene, *GAPDH*, or to the optimal reference gene identified in this study, *TBP*. *VEGFA* mRNA was significantly upregulated by 136% after 24 h of incubation of A549 cells in HX when normalized to *TBP*; however, this effect was diminished to a mere, non-significant 9% when *GAPDH* was used as a reference gene (Figure 4a). In EA.hy926 cells, 72 h of HX caused a significant upregulation of *VEGFA* mRNA expression by 380 ± 46% when normalized to *TBP* (*p* < 0.01); however, when normalized to *GAPDH*, *VEGFA* expression was not significantly changed (+410 ± 430%; n.s.) (Figure 4b).

## 3. Discussion

Quantitative real-time polymerase chain reaction (qRT-PCR) has become a widely used, standard laboratory procedure to quantify gene expression at the mRNA level. This method relies on the comparison of the mRNA expression level of the target gene with that of a stably expressed reference gene. Many research groups choose the reference gene based upon reports in the literature generated by other groups and, possibly, in slightly different experimental conditions. However, it has been shown that many frequently used reference genes may not be as stably expressed under certain experimental conditions as is believed. In a systematic review of reference gene expression studies, Chapman and Waldenström [18] found that whilst many studies used *GAPDH* or *ACTB* as endogenous controls, only few studies amongst those in which a panel of putative reference genes was tested found *ACTB* or *GAPDH* to be optimal. For example, *GAPDH* is known to be stimulated by diverse biological factors including glucose, insulin, oxidative stress, and hypoxia [19]. Likewise, mRNA expression of *ACTB* may be modulated in multiple biological conditions including hypoxia [20,21].

In the present study, we analyzed nine putative reference genes for the stability of their expression after 24 h and 72 h of incubation at 1% O_2_ (HX) as compared to NX (21% O_2_) in four cell lines from the cardiopulmonary system. We used the immortalized cell lines EA.hy926 derived fb rom human umbilical vein endothelial cells, as they are a frequently used cell line representing endothelial cells, and A549, an immortalized cell line derived from human airway epithelium, as well as primary human coronary endothelial cells (HCAECs) and pulmonary arterial endothelial cells (HPAECs). This allowed us to analyze the variability brought about by experimental condition (i.e., HX vs. NX) and by cell line. As our initial analysis showed that hypoxia-induced variability in reference gene expression showed minor differences between 24 h and 72 h of HX, we performed all further analyses by combining expression values for 24 h and 72 h of hypoxia versus normoxia. 

As expected, there was a wide range of variability in stability values between the nine putative reference genes in each of the four methods of stability analysis that we applied. The coefficient of variation of the Ct value showed the highest variability for *18S*, *B2M*, *ACTB*, *SDHA*, and *GAPDH*, whilst *TBP*, *PPIA*, and *RPLP1* had the smallest CV Ct values in almost all cell lines.

Reference genes should be expressed in a similar copy number to the gene of interest [22] to allow for optimal quantitation. The Ct value of a suitable reference gene should be between 15 and 30, and the SD should not surpass 1.0 [23,24]. However, as rRNA accounts for about 80% of total cellular RNA, rRNA transcripts like *18S* tend to be much more abundant than mRNA transcripts. Therefore, even small changes in *18S* would result in large, apparent differences in target gene expression after normalization. In addition, rRNA and mRNA are transcribed by different RNA polymerases (polymerase I and polymerase II, respectively). Thus, their synthesis is regulated via different pathways [18]. This is why *18S* should not be the reference gene of first choice.

Pairwise comparison of the standard deviation of the Ct values in the experimental conditions tested has been described as another method to compare the suitability of different putative reference genes [17]. Pairwise comparison of all possible combinations of reference genes allows to calculate the mean SD for each gene; the gene with the lowest SD is supposed to be the best suited reference gene for the experimental conditions chosen. In our study, *GAPDH* and *SDHA* showed the highest mean SD values, whilst *TBP* and *RPLP1* had the lowest SD values in almost all cell lines. *PPIA*, which had low mean SD values in most cell lines, performed poorly in A549 cells.

We next analyzed expression stability using the NormFinder algorithm. *18S*, *B2M*, and *TBP* were the genes for which stability results were closest in all four cell lines, whilst all other genes showed large variation in the stability values between cell lines. A similar approach had been chosen by Foldager et al. [25] who received small stability values for *RPL13A* and *18S* in chondrocytes, thereby indicating higher stability in comparison to *GAPDH*, *B2M* and *ACTB*.

Algorithms like NormFinder rank candidate reference genes by order of increased stability. However, different methods and algorithms might lead to different rankings [26], and there is no objective criterion to choose the most appropriate analysis for reference gene selection. In line with this, we found in our study that the three different approaches led to different rankings in the four cell lines. Therefore, we performed multiple analyses including Ct CV, pairwise DeltaCt, and the NormFinder analysis, and combined the results of these analyses in a final sum rank.

Although various studies have shown that the expression of one of the most commonly used reference genes, *GAPDH*, is differentially regulated in various experimental conditions including hypoxia, its expression stability in a specific experimental setup is often not checked prior to gene expression studies. *GAPDH* has been described to be upregulated in endothelial cells during hypoxia [10,27], which may be explained by its enzymatic function in anaerobic glycolysis [28].

Our present study shows that not only *GAPDH* shows poor expression stability during hypoxia: *SDHA*, a gene encoding for one subunit of the mitochondrial succinate dehydrogenase, which is involved in mitochondrial energy metabolism, β-actin, forming part of the cytoskeleton, and β2-microglobulin, a component of the major histocompatibility complex class I, also showed poor ranking in our analyses. By contrast, the ribosomal proteins, *RPLP1* and *RPL13A*, the protein folding modulator, *PPIA*, and the transcription initiator, *TBP*, generally showed good stability of expression in hypoxia throughout almost all analyses, which granted good rankings for these genes.

Variability in gene expression is not only brought about by experimental conditions, but also by the cell lines included in a specific experiment. Therefore, we tested which reference gene(s) might be preferable when comparing different sets of cell lines. Whilst the overall analysis of all cell lines showed that *TBP* was the top-ranking gene, followed by *PPIA* and *RPLP1*, for the comparison of arterial endothelial cells from the systemic versus pulmonary circulation, *RPLP1* and *TBP* ranked best. By contrast, when comparing the response of pulmonary epithelial and endothelial cells to hypoxia, *TBP*, *PPIA*, and *RPL13A* were more stably expressed in these cell lines and thus the best choice as reference genes, and comparison of primary human endothelial cell gene expression in the systemic versus pulmonary circulation, i.e., the comparison of HCAECs and HPAECs, *B2M* and *RPLP1* showed the best stability of expression. These findings underscore the recommendation that suitability of reference genes should be tested for each set of biological experiments comprising mRNA expression studies in the specific in vitro model chosen.

Finally, our data also show the possible consequences arising from inadequate choice of the reference gene. Whilst it is well known and has been documented multiple times that *VEGFA* is upregulated in hypoxia, an effect that is mediated by hypoxia-inducible factor-1α (HIF1α) [29] choosing an unstably expressed reference gene may hamper detection of relevant changes in gene expression in hypoxia. If the reference gene is upregulated in hypoxia, upregulation of the target gene may be obscured when the expression ratio is calculated as part of the ΔΔCt method in qRT-PCR. We show here that, even when using the identical biological samples, mRNA expression analysis of *VEGFA* in A549 cells normalized to either *GAPDH* or *TBP* as reference genes results in “no change” or a significant 136 % increase in expression, depending on which of the two reference genes was used. In addition, large variability in the expression of the chosen reference gene may conceal significant increases in *VEGFA* expression by rendering results insignificant, as evidenced by the high variability of gene expression normalized to *GAPDH* in EA.hy926 cells after 72 h of exposure to hypoxia. This example underscores why choosing an appropriate, stable reference gene is necessary prior to analyzing relative gene expression.

## 4. Materials and Methods

### 4.1. Cell Culture

The stable human alveolar epithelial cell line A549 (RRID: CVCL_0023) and human endothelial cell line EA.hy926 (RRID: CVCL_3901; both ATCC, Manassas, VA, USA), primary human coronary artery endothelial cells (HCAECs; Promocell, Heidelberg, Germany), and primary human pulmonary artery endothelial cells (HPAECs; Promocell, Heidelberg, Germany) were used in this study. EA.hy926 and A549 cells were cultured in DMEM supplemented with 10% FCS (Capricorn Scientific, Ebsdorfergrund, Germany) and a final concentration of 100 U/mL penicillin, 100 μg/mL streptomycin (Merck, Darmstadt, Germany), at 37 °C and 7% or 5% CO_2_, respectively.

HPAECs were cultured in Endothelial Cell Growth Medium (Promocell, Heidelberg, Germany), a low-serum culture medium for endothelial cells from large blood vessels; HCAECs were grown in Endothelial Cell Growth Medium MV (Promocell, Heidelberg, Germany), which is suitable for endothelial cells from the coronary artery. For subculturing, a DetachKit (Promocell, Heidelberg, Germany) was used according to the manufacturer’s instructions. Briefly, culture medium was aspirated carefully and cells were washed once with HEPES BSS. Subsequently, 5 mL of a 0.04% trypsin/0.03% EDTA solution were added. Cell detachment was microscopically monitored and 5 mL of the trypsin neutralization agent (Promocell) were added when cells began to detach from the culture vessel surface. Cells were pelleted by centrifugation (220× *g*) and re-suspended in culture medium. Cells were maintained at 37 °C and 5% CO_2_. All experiments with primary cells were performed in passages 6–7 to ensure optimal viability and activity of the cells.

### 4.2. Experimental Conditions (Hypoxia Versus Normoxia)

For all experiments, cell culture medium was pre-conditioned for 24 h in a hypoxia chamber (1% O_2_, 37 °C) or the normoxia chamber (21% O_2_, 37 °C), respectively. Cells were seeded 24 h before incubation in an appropriate density to reach 70–80% confluence after the particular incubation period, and pre-conditioned medium was added at time point zero. Medium was replaced every 24 h to ensure optimal nutrient availability. Cells were harvested after 24 h or 72 h of hypoxia, respectively (HX; 1% O_2_). Cells grown in normoxic conditions (NX; 21% O_2_) were used as controls. All experiments were performed as six biological replicates for each condition.

### 4.3. RNA Isolation and Reverse Transcription

After harvesting, cells were immediately transferred to 1 mL of prechilled Trizol (ThermoFisher Scientific, Waltham, MA, USA) and kept at −80 °C until further processing. Sample clean-up and on column removal of genomic DNA (gDNA) were performed using PureLink™ RNA Mini Kit in combination with PureLink™ DNase (both ThermoFisher). RNA integrity and complete gDNA digestion were verified by agarose gel electrophoresis; RNA samples were stored at −80 °C until further use. A total of 2 µg of total RNA were reverse-transcribed with MultiScribe™ High Capacity cDNA Reverse Transcriptase (ThermoFisher). The final reaction mix contained 2 µg of total RNA, 0.8 µL 100 mM dNTP-Mix (final concentration, 5 mM), 2 µL 10× random primers, 1 µL MultiScribe™ reverse transcriptase (50 U/mL), and 2 µL 10× RT buffer and nuclease-free water to adjust the reaction volume to 20 µL. Reverse transcription was performed according to the following protocol: primer annealing: 10 min at 25 °C; reverse transcription: 120 min at 37 °C; enzyme deactivation: 5 min at 85 °C, cool-down to 4 °C.

### 4.4. Quantitative Real-Time PCR

Quantitative real-time PCR (qRT-PCR) was performed in a total volume of 10 µL using 18 ng cDNA, Taqman Fast Advanced Master Mix, and gene-specific Taqman™ assays containing unlabeled gene-specific primers and 5′-FAM TaqMan™ MGB probe with 3′-nonfluorescent quencher (all ThermoFisher). The following housekeeping genes were analyzed: *ACTB*, *B2M*, *GAPDH*, *18S*, *TBP*, *SDHA*, *PPIA*, *RPLP1*, and *RPL13A*. The spelled out gene names and the specific assays used for their quantification are listed in Table S1. These candidate genes had been selected based on the following criteria: (a) classical, often used internal control genes, (b) genes used for normalization in other studies investigating the influence of hypoxia on gene expression. All samples were prepared as technical triplicates; non-template controls were included for each assay. The qRT-PCR reaction was run on a Quantstudio 5 System (ThermoFisher) using the following cycling conditions: UNG incubation: 2 min at 50 °C; activation 10 min at 95 °C, 40 cycles of denaturation (15 sec at 95 °C) and annealing/extension (1 min at 60 °C). Each biological replicate (n = 6 per condition) was run as technical triplicate in the PCR reaction; mean Ct values from technical triplicates were used to analyze gene expression for each biological replicate. All qRT-PCR reactions of one gene and cell line were performed in the same run.

### 4.5. Calculation of Gene Expression Stability in Hypoxia and Normoxia and Statistical Analysis

To analyze expression stability of the selected candidate reference genes in normoxia versus hypoxia, different methods were sequentially applied: In a first step, mean cycle threshold (Ct) values and its coefficient of variation (Ct CV) were calculated for each putative reference gene in each of the cell lines, including normoxia and hypoxia (24 h and 72 h) as experimental conditions. Thus, for each gene, PCR results (mean Ct values of 3 technical replicates) were included from 3 experimental conditions (NX, HX 24 h, HX 72 h) which were each measured in 6 biological replicates. Genes were then ranked according to Ct CVs within each cell line. In a second step, this analysis was repeated for all cell lines together and for different combinations of two cell lines each, to show variation according to cell lines and variation according to experimental conditions.

In the next step, the expression stability of the candidate reference genes was analyzed by pairwise comparison of ΔCt values between all combinations of two candidate genes according to Silver et al. [17]. For this, mean Ct values were calculated for NX and HX (24 h and 72 h) for each gene in each cell line. Then, pairs of two genes each were compared amongst the nine putative reference genes within each cell line, and differences of Ct values for each gene were calculated with each other gene (i.e., eight differences in Ct values for each gene). The mean SD of these differences were noted, and genes were ranked according to these expression stability values.

In a fourth step, the freely available NormFinder Excel add-in [30] was used to analyze the expression stability of ΔCt values based on biological groups (NX, HX 24 h and HX 72 h) for each cell line separately as well as for all cell lines together. NormFinder uses a more complex algorithm as compared to indices of overall expression variation, in that helps to overcome the circular problem of evaluating the expression stability of a putative reference gene in the absence of a reliable reference measure by using a model-based approach that has been developed and validated in detail by others [30]. The NormFinder algorithm takes into account both, overall expression variation as well as variation in gene expression between the biological subgroups for the putative reference genes.

As each of the previously described analytical methods to assess reference gene expression stability has been based on a different mathematical algorithm, it remains hard to decide which single algorithm provides the most reliable result. We hypothesized that the most stable expression of a reference gene would be found for a gene that ranks high across all analyses. Therefore, a final mean rank was calculated for each gene by calculating the rank sum of each gene’s ranks with every analytical method. Rank sums are provided for analysis of all four cell types, as well for sets of two cell types (see Supplementary Table S2).

Finally, we compared the relative gene expression of VEGFA in hypoxia versus normoxia after normalization to GAPDH, a commonly used reference gene, and TBP, which showed good expression stability in all cell lines during hypoxia, to exemplify the impact of reference gene selection on the outcome of biological experiments in hypoxia. *VEGFA* mRNA expression determined using the ΔΔCt method [31].

All data are presented as mean ± standard deviation (SD) unless specified differently. Differences between two biological groups were tested for statistical significance by using the Mann-Whitney U-test; comparisons of more than two groups or time points were assessed by ANOVA with adjustment of *p* for multiple testing (Dunnett’s multiple comparisons test). All calculations were performed using R statistical package version 4.4.1 Tidyverse and GraphPad Prism version 6.01 (GraphPad Software, San Diego, CA, USA). A *p* < 0.05 was considered significant in all cases.

## 5. Conclusions

In conclusion, while qRT-PCR is a frequently used method for quantification of mRNA expression of genes, diligent choice of the reference gene and experimental validation of its suitability in the cell type and experimental condition chosen is vital for the generation of valid experimental results. Hypoxia is a strong modulator of cellular processes ranging from energy metabolism to cell growth and beyond; therefore, this experimental condition requires specific care in reference gene selection. The optimal reference gene may vary depending on the cell lines compared, and thus needs to be defined for each experimental setting.

## Figures and Tables

**Table 1:** Coefficients of variation for Ct values in normoxia and hypoxia (24 h and 72 h) for EA.hy926 cells, A549 cells, HCAECs, HPAECs, and for all cell lines combined.

EA.hy926		A549		HCAECs		HPAECs		All Cell Lines	
Gene/Ct CV [%]		Gene/Ct CV [%]		Gene/Ct CV [%]		Gene/Ct CV [%]		Gene/Ct CV [%]	
*TBP*	1.48	*RPL13A*	1.14	*TBP*	1.13	*SDHA*	0.71	*PPIA*	1.39
*B2M*	1.86	*SDHA*	1.20	*PPIA*	1.19	*ACTB*	0.77	*TBP*	1.62
*RPLP1*	2.21	*RPLP1*	1.41	*RPL13A*	1.27	*B2M*	0.81	*ACTB*	1.72
*SDHA*	2.21	*GAPDH*	1.42	*RPLP1*	1.43	*TBP*	0.99	*RPLP1*	2.27
*RPL13A*	2.33	*TBP*	1.46	*B2M*	1.70	*GAPDH*	1.03	*RPL13A*	2.33
*PPIA*	2.38	*ACTB*	1.64	*ACTB*	1.81	*PPIA*	1.05	*GAPDH*	2.78
*GAPDH*	2.66	*B2M*	1.79	*GAPDH*	1.98	*RPL13A*	1.20	*SDHA*	2.93
*ACTB*	4.41	*PPIA*	1.85	*SDHA*	4.19	*RPLP1*	1.37	*B2M*	3.55
*18S*	7.77	*18S*	4.05	*18S*	4.73	*18S*	3.46	*18S*	4.72

Gene names are listed in order of increasing coefficients of variation for each cell line.

**Table 2:** Ranking of reference genes according to mean SD values after pairwise comparison of ΔCt values.

Rank	EA.hy926		A549		HCAECs		HPAECs		All Cell Lines	
	Gene/Mean SD		Gene/Mean SD		Gene/Mean SD		Gene/Mean SD		Gene/Mean SD	
1	*B2M*	0.471	*RPL13A*	0.265	*TBP*	0.305	*PPIA*	0.196	*PPIA*	0.590
2	*TBP*	0.530	*ACTB*	0.276	*RPLP1*	0.310	*B2M*	0.209	*TBP*	0.604
3	*RPLP1*	0.555	*B2M*	0.285	*PPIA*	0.341	*18S*	0.218	*RPLP1*	0.615
4	*SDHA*	0.572	*TBP*	0.290	*RPL13A*	0.347	*ACTB*	0.218	*RPL13A*	0.632
5	*RPL13A*	0.587	*RPLP1*	0.290	*ACTB*	0.352	*RPLP1*	0.226	*18S*	0.679
6	*PPIA*	0.615	*18S*	0.298	*18S*	0.368	*GAPDH*	0.226	*ACTB*	0.742
7	*18S*	0.649	*SDHA*	0.306	*B2M*	0.375	*TBP*	0.239	*GAPDH*	0.752
8	*GAPDH*	0.711	*PPIA*	0.348	*GAPDH*	0.460	*RPL13A*	0.340	*SDHA*	0.913
9	*ACTB*	0.764	*GAPDH*	0.379	*SDHA*	0.976	*SDHA*	0.372	*B2M*	1.003

Gene names are listed in order of increasing mean SD for each cell line.

**Table 3:** Final ranking of all reference genes.

EA.hy926		A549		HCAECs		HPAECs		All Cell Lines	
Gene	Score	Gene	Score	Gene	Score	Gene	Score	Gene	Score
*B2M*	4	*RPL13A*	4	*PPIA*	7	*PPIA*	8	*TBP*	8
*TBP*	5	*ACTB*	9	*RPLP1*	7	*ACTB*	9	*PPIA*	9
*RPLP1*	11	*RPLP1*	11	*TBP*	8	*B2M*	9	*RPLP1*	9
*SDHA*	11	*B2M*	14	*RPL13A*	11	*18S*	14	*RPL13A*	12
*PPIA*	15	*SDHA*	15	*ACTB*	14	*GAPDH*	17	*18S*	16
*RPL13A*	18	*TBP*	16	*B2M*	19	*SDHA*	19	*B2M*	18
*GAPDH*	19	*18S*	20	*18S*	20	*TBP*	19	*ACTB*	20
*18S*	23	*GAPDH*	22	*GAPDH*	23	*RPL13A*	20	*GAPDH*	20
*ACTB*	26	*PPIA*	24	*SDHA*	26	*RPLP1*	20	*SDHA*	23

Gene names are listed in the order of their final ranking in each analysis and in alphabetical order when rank scores are identical.

**Table 4:** Ranking of reference genes for three sets of cell line combinations.

HCAECs vs. EA.hy926		HCAECs vs. HPAECs		HPAECs vs. A549	
Gene	Score	Gene	Score	Gene	Score
*RPLP1*	4	*B2M*	6	*TBP*	9
*TBP*	8	*RPLP1*	6	*PPIA*	10
*RPL13A*	11	*PPIA*	11	*RPL13A*	10
*PPIA*	12	*TBP*	11	*ACTB*	11
*18S*	18	*ACTB*	17	*SDHA*	14
*B2M*	18	*18S*	18	*18S*	15
*GAPDH*	18	*GAPDH*	20	*B2M*	21
*ACTB*	22	*RPL13A*	20	*GAPDH*	22
*SDHA*	24	*SDHA*	26	*RPLP1*	23

Gene names are listed in the order of their final ranking in each analysis.

## Data Availability

The data generated in this study are available in this article and in the Supplementary Tables provided.

## Supplementary Materials

The following supporting information can be downloaded at: https://www.mdpi.com/article/10.3390/ijms26041763/s1.
