# Supplementary material for: Selection of Reference Genes for Normalization of Gene Expression After Exposure of Human Endothelial and Epithelial Cells to Hypoxia
Source: Int J Mol Sci. 2025 Feb 19;26(4):1763. doi: 10.3390/ijms26041763 (PMC11855034; doi:10.3390/ijms26041763)
Supplement: Supplementary file 1 [file ijms-26-01763-s001.zip › ijms-3466337-supplementary.pdf]

Selection of reference genes for normalization of gene expression after exposure of human endothelial and epithelial cells to hypoxia.

Juliane Hannemann, Lena Schmidt-Hutten, Jannik Hannemann, Fiona Kleinsang, Rainer Böger

Supplementary Material

Table S1. Overview of candidate reference genes used in this study.

Gene abbreviation	Gene name	Molecular function	Assay ID
ACTB	Actin beta	Cytoskeletal component	Hs01060665_g1
B2M	Beta-2-microglobulin	Component of major histocompatibility complex	Hs00187842_m1
GAPDH	Glyceraldehyde-3-phosphate dehydrogenase	Glycolysis	Hs00266705_g1
18S	18S ribosomal RNA	Part of ribosomal RNA	Hs03003631_g1
TBP	TATA-box binding protein	Initiation of transcription	Hs00427620_m1
SDHA	Succinate dehydrogenase complex flavoprotein subunit A	Glycolysis	Hs00188166_m1
PPIA	Peptidylprolyl isomerase A	Protein folding	Hs04194521_s1
RPLP1	Ribosomal protein lateral stalk subunit P1	Ribosomal phosphoprotein	Hs01653088_g1
RPL13a	Ribosomal protein L13a	Enabling mRNA binding activity	Hs04194366_g1

Assay IDs refer to TaqMan assays from Thermo Fischer Scientific that were used in this study.

Table S2. Ranking of putative reference genes for direct comparisons of pairs of cell types.

EA.hy926 cells versus HCAEC				HCAEC versus HPAEC				HPAEC versus A549 cells			
Ct CV	Pairwise ΔCt	Norm Finder	Total Score	Ct CV	Pairwise ΔCt	Norm Finder	Total Score	Ct CV	Pairwise ΔCt	Norm Finder	Total Score
TBP	*RPLP1*	*RPLP1*	*RPLP1* (4)	*PPIA*	*RPLP1*	*RPLP1*	*RPLP1* (6)	*SDHA*	*TBP*	*RPL13A*	*TBP* (9)
RPLP1	*PPIA*	*B2M*	*TBP* (8)	*B2M*	*B2M*	*B2M*	*B2M* (6)	*TBP*	*PPIA*	*18S*	*PPIA* (10)
RPL13A	*RPL13A*	*TBP*	*RPL13A* (11)	*ACTB*	*TBP*	*TBP*	*TBP* (11)	*PPIA*	*ACTB*	*ACTB*	*RPL13A* (10)
PPIA	*TBP*	*18S*	*PPIA* (12)	*RPLP1*	*PPIA*	*18S*	*PPIA* (11)	*RPL13A*	*18S*	*B2M*	*ACTB* (11)
GAPDH	*18S*	*RPL13A*	*B2M* (18)	*TBP*	*18S*	*RPL13A*	*ACTB* (17)	*ACTB*	*RPL13A*	*PPIA*	*SDHA* (14)
SDHA	*GAPDH*	*PPIA*	*GAPDH* (18)	*GAPDH*	*ACTB*	*PPIA*	*18S* (18)	*GAPDH*	*SDHA*	*TBP*	*18S* (15)
ACTB	*ACTB*	*GAPDH*	*18S* (18)	*RPL13A*	*GAPDH*	*GAPDH*	*RPL13A* (20)	*RPLP1*	*RPLP1*	*SDHA*	*B2M* (21)
B2M	*B2M*	*ACTB*	*ACTB* (22)	*SDHA*	*RPL13A*	*ACTB*	*GAPDH* (20)	*B2M*	*GAPDH*	*GAPDH*	*GAPDH* (22)
18S	*SDHA*	*SDHA*	*SDHA* (24)	*18S*	*SDHA*	*SDHA*	*SDHA* (26)	*18S*	*B2M*	*RPLP1*	*RPLP1* (23)

In each row, genes are listed according to the rank in each of the analyses, so that the gene listed in line 1 has the highest rank, whereas the gene listed in line 9 has the lowest rank. The total score is the sum of the rank scores of all three tests for each gene in the direct comparison of the two cell types as indicated.
